# Characterization and Structure–Property Relationships of Organic–Inorganic Hybrid Composites Based on Aluminum–Magnesium Hydroxycarbonate and Azo Chromophore

**DOI:** 10.3390/molecules24050880

**Published:** 2019-03-01

**Authors:** Anna Marzec, Bolesław Szadkowski, Jacek Rogowski, Waldemar Maniukiewicz, Marian Zaborski

**Affiliations:** 1 Institute of Polymer and Dye Technology, Faculty of Chemistry, Lodz University of Technology, Stefanowskiego 12/16, 90-924 Lodz, Poland; boleslaw.szadkowski@edu.p.lodz.pl (B.S.); marian.zaborski@p.lodz.pl (M.Z.); 2 Institute of General and Ecological Chemistry, Lodz University of Technology, Zeromskiego 116, 90-924 Lodz, Poland; jacek.rogowski@p.lodz.pl (J.R.); waldemar.maniukiewicz@p.lodz.pl (W.M.)

**Keywords:** hybrid pigment, azo dye, aluminum–magnesium hydroxycarbonate, organic–inorganic composite, pigment stability

## Abstract

In this study, novel organic–inorganic composites were prepared by the complexation of dicarboxylic azo dye (AD) with aluminum–magnesium hydroxycarbonate (AlMg–LH). This procedure provides an effective method for the stabilization of dicarboxylic organic chromophores on an AlMg−LH host. The structures of the hybrid composites were examined by X-ray diffraction (XRD), secondary ion mass spectrometry (TOF-SIMS), 27-Al solid-state nuclear magnetic resonance (NMR) spectroscopy, thermogravimetric analysis (TGA) and scanning transmission electron microscopy (STEM). The TOF-SIMS method was applied to investigate the metal–dye interactions and to monitor the thermal stability of the organic–inorganic complexes. Secondary ion mass spectrometry confirmed the presence of a characteristic peak for C_18_H_10_O_5_N_2_Mg_2_^2+^, indicating that both carboxylic groups interacted with AlMg−LH by forming complexes with two Mg^2+^ ions. Modification with hybrid pigments affected the crystal structure of the AlMg−LH mineral, as shown by the appearance of new peaks on the X-ray diffraction patterns. Adsorption of the dicarboxylic chromophore not only led to significantly enhanced solvent resistance but also improved the thermal and photostability of the hybrid pigments. We propose a possible arrangement of the azo dye in the inorganic matrix, as well as the presumed mechanism of stabilization.

## 1. Introduction

Azo dyes are an important class of modern dyes and are extensively used in textiles, leather, printing, food, drugs, and optical devices. However, some azo dyes have a limited range of applications, for example, due to their low melting points, high tendency to migrate, and poor thermo- or/and photostability, some dyes cannot be used in polymer materials. Most of these disadvantages can be overcome by complexation of the organic chromophores with inorganic hosts [1,2]. Recently, there has been great interest in multifunctional hybrid materials, which combine the advantages of both organic materials (light weight, versatility, intense color) and inorganic materials (high thermal and chemical resistance) [3,4]. Such hybrid composites may also exhibit special physicochemical properties, which are not present in the separate components [5,6]. Various procedures have been proposed for obtaining chemically stable pigments. Numerous studies have focused on sol-gel methods, and on the adsorption of organic chromophores onto an inorganic host [7,8]. Organic–inorganic materials can also be produced by the complexation of natural and synthetic dye molecules with solid host matrices. Inorganic host matrixes such as gamma alumina, silica, zeolites, and layered double salts can improve the thermal-, photo-, and chemical stability of dyes [9,10,11,12,13,14,15,16,17,18].

Guillermin et al. prepared hybrid materials based on montmorillonite, a cationic polymer, and carminic acid [19]. They suggest that the adsorption mechanism may be related to the OH groups of the carminic acid and in both surface groups present on the edge faces of montmorillonite bonding with adsorbed cationic polymers molecules on the basal faces. These authors propose different methods for studying the interactions between the organic and inorganic parts, including infrared (IR) and nuclear magnetic resonance (NMR) spectroscopies. Ramirez et al. describe the synthesis of hybrid pigments by the combination of natural chromophores, such as carminic acid, alizarin, purpurin, curcumin, fluorescein, and betacyanins with a gamma alumina matrix (γ-Al_2_O_3_) [20]. They also used spectroscopic techniques to confirm the formation of aluminum complexes between coordinative unsaturated sites in the aluminum and some organic groups (carboxylic acid, quaternary ammonium, and β-keto enol) present in the dye chromophores. Girdthep et al. produced natural lake pigments based on Sappan and kaolinite mineral [21]. The enhanced thermal stability of the hybrid pigment was attributed to electrostatic attraction between the Al(III) ion, the kaolinite surface, and brazilein molecules. Recently, several works have attempted the intercalation of various azo dyes into layered aluminum–magnesium (or zinc–aluminum) hydroxycarbonate structures [22,23,24,25,26,27]. However, the intercalation of dye into the interlayer space of aluminum–magnesium hydroxycarbonate is time-consuming and requires an inert atmosphere [28,29,30]. Therefore, the precipitation method still represents an important method of stabilization of organic–inorganic hybrids.

The purpose of this study was to investigate the synthesis, characterization, and application of a red pigment obtained by the precipitation of azo dye onto the AlMg−LH matrix. The precipitation method has been shown to lead to the formation of metal–dye complexes characterized by high insolubility, good color strength, and improved technical performance in applications [31,32]. Due to the presence of two carboxylic groups, the selected azo chromophore was expected to have an affinity for the inorganic component, which is basic in nature. The morphology of the MgAl−LH before and after the incorporation of the guest dye was characterized by X-ray powder diffraction (XRD) and scanning electron microscopy (SEM/STEM). The organic–inorganic hybrid was also examined for thermal stability (TGA), solvent resistance, and photostability. TOF-SIMS and ^27^Al NMR spectroscopy were used to investigate the interactions between the azo dye and particular ions present in the AlMg−LH structure.

## 2. Results and Discussion

### 2.1. Secondary Ion Mass Spectrometry (TOF-SIMS)

**Figure 1:**
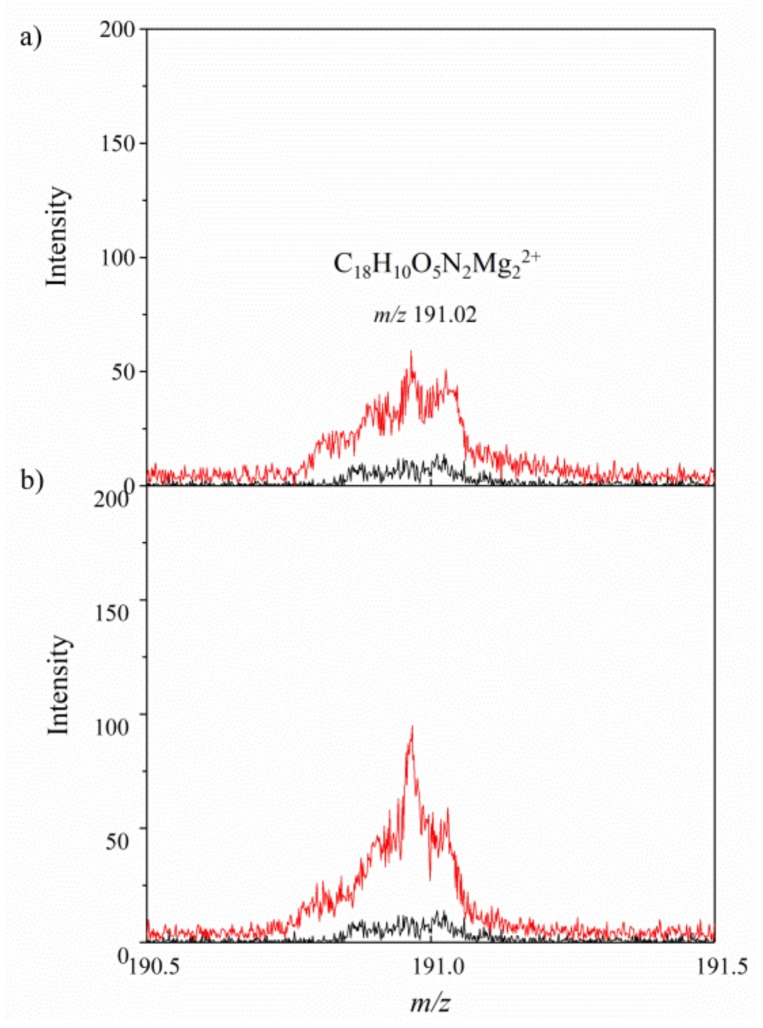
Positive secondary ion mass spectrometry TOF-SIMS spectra for aluminum–magnesium hydroxycarbonate AlMg−LH (black line) and AlMg−LH /AD 10% (red line) (**a**) and AlMg−LH (black line) and AlMg−LH /AD 20% (red line) (**b**) samples.

To better understand the stabilization mechanism of the organic–inorganic hybrid, the possible interactions between the dicarboxylic azo dye (AD) and particular ions present in the AlMg−LH layers were investigated using the TOF-SIMS method. In our previous work, we studied the interactions between alizarin and aluminum–magnesium hydroxycarbonates with different Mg/Al ratios [33]. The results of TOF-SIMS experiments confirmed the formation of alizarin complexes with both Mg^2+^ and Al^3+^ ions in the inorganic hosts. In the current study, it was found that the addition of AD dye to AlMg−LH resulted in the appearance of a characteristic peak at *m/z* 191 in the TOF-SIMS spectrum, which was not observed in the spectrum of the AlMg−LH sample (Figure 1a). This peak was assigned to the C_18_H_10_O_5_N_2_Mg_2_^2+^ ion, indicating interaction between the azo dye and the two magnesium ions in the AlMg−LH/AD 10% matrix, whereas C_18_H_11_O_5_N_2_Mg^+^ containing only one Mg atoms was not present in the AlMg−LH/AD 10% spectrum. Moreover, comparison of the TOF-SIMS spectra in Figure 1a,b demonstrates that the intensity of the C_18_H_10_O_5_N_2_Mg_2_^2+^ peak grew as the concentration of azo dye in the inorganic matrix increased. We concluded that the azo-based colorants were stabilized by interactions with Mg^2+^ ions. In contrast to anthraquinone-based colorants [33], the TOF-SIMS spectra of the AlMg−LH/AD 10% and AlMg−LH/AD 20% samples did not contain peaks corresponding to complex of dye with Al^3+^ ions. These observations may indicate that the complex formed by the dye and Al was not stable enough to be emitted from the sample as a non-fragmented ion, but this does not exclude the possibility of interaction between Al^3+^ and dye in the LH matrix. The presence of some slight interactions was observed by NMR studies.

**Figure 2:**
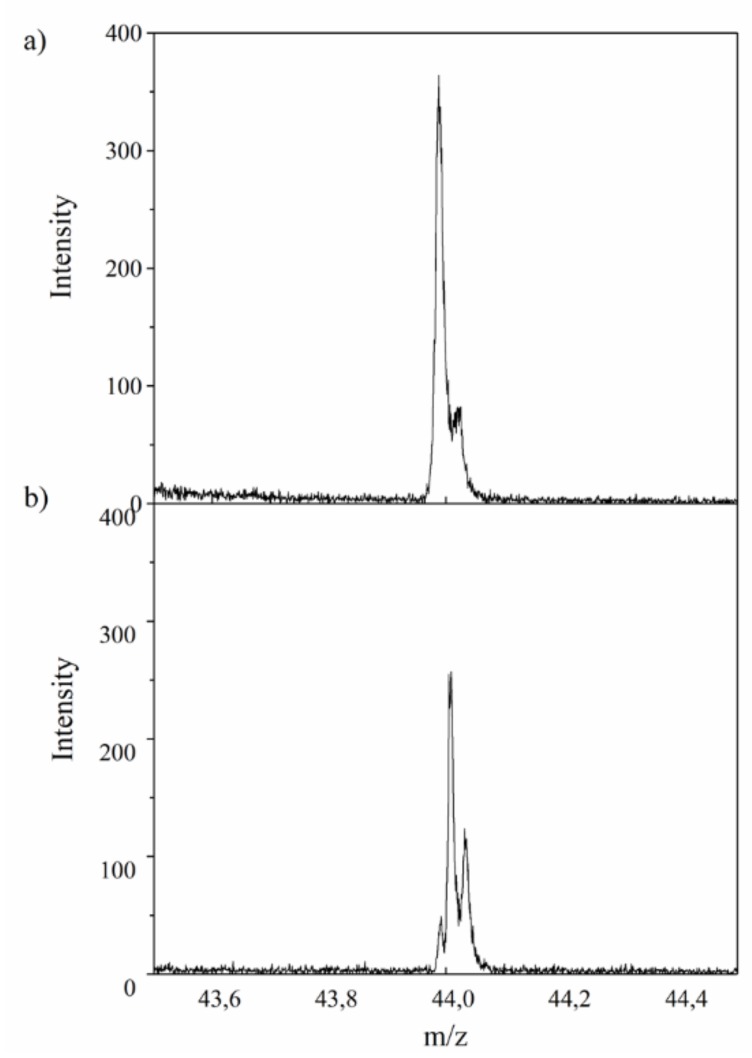
TOF-SIMS spectra of CO_2_^−^ ions emitted from (**a**) AlMg−LH and (**b**) AlMg−LH/AD 10% samples.

It is worth noting that the modification of AlMg−LH with AD was accompanied by a decrease in the emission of CO_2_^−^ ions, indicating the presence of carbonate ions (CO_3_^2−^) in the sample (Figure 2). This reduction in the emission of CO_2_^−^ ions from the AlMg−LH/AD pigment was most likely related to the fact that CO_3_^2−^ ions were partially removed from the AlMg−LH/AD, as a result of the competitive interaction of C_18_H_10_O_5_N_2_^−^ ions with Mg^2+^ ions in the AlMg−LH structure. This observation is in line with the results of further TGA studies.

Numerous reports based on NMR spectroscopy, most often of ^13^C and ^27^Al MAS NMR spectra, have suggested that organic dyes form complexes with clay minerals [20,34]. However, this method is restricted to the analysis of dye interactions involving only a limited number of ions, and does not involve ions such Mg^2+^ or Zn^2+^. TOF-SIMS may be considered as a complementary technique, providing insight into the mechanism of stabilization by metal mixed oxides of carboxylic chromophores.

### 2.2. Solid State Nuclear Magnetic Resonance of ^27^Al (MAS NMR)

**Figure 3:**
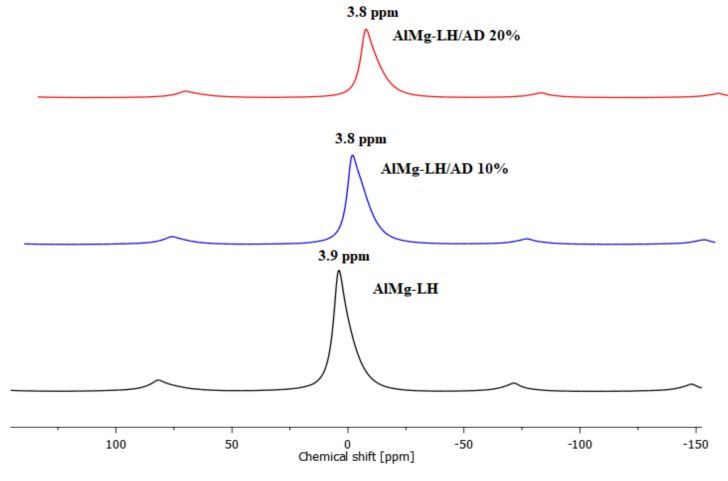
Solid state nuclear magnetic resonance ^27^Al MAS NMR spectra for AlMg−LH, AlMg−LH/AD 10%, and AlMg−LH/AD 20%.

^27^Al MAS NMR spectra of AlMg−LH before and after the adsorption of the azo dye are depicted in Figure 3. ^27^Al resonance line positions are very sensitive to coordination number and expected to occupy the −5 to 15 ppm range for AlO_6_ sites [35]. Spectrum of the AlMg−LH sample before complexation shows a relatively narrow resonance peak at a chemical shift, δ, of ~3.9 ppm, which represents the octahedral coordination of Al. The NMR signal of the hybrid pigment was very close to that of the unmodified AlMg−LH sample. The addition of the azo chromophore to the AlMg−LH host decreased the relative intensity of the resonance peak. We also noted a chemical shift (from 3.9 to 3.8 ppm) in both the case of AlMg−LH/AD 10% and AlMg−LH/AD 20% (Figure 3). A similar shift has been reported for Al in montmorillonite modified with organic dyes [36]. Therefore, we conclude that the changes we observed in the NMR spectra may suggest the presence of some interactions between the Al ions and the dye in the samples.

### 2.3. X-ray Diffraction Study (XRD)

**Figure 4:**
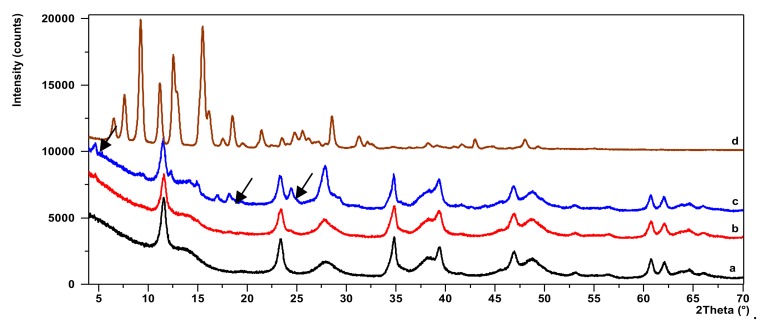
Diffraction patterns for AlMg−LH (**a**), AlMg−LH/AD 10% (**b**), AlMg−LH/AD 20% (**c**), and azo dye (AD) (**d**).

Figure 4 shows X-ray powder diffraction patterns for AlMg−LH, AlMg−LH/AD 10%, AlMg−LH/AD 20%, and AD. The diffraction pattern of pure AlMg−LH in Figure 4a consisted of three sharp peaks at a low 2*θ* angle, equivalent to diffraction by planes (003), (006), and (009). This indicates good AlMg−LH crystallinity [37]. The interlayer distances of (*d*_003_) and (*d*_006_), corresponding to 0.758 nm and 0.381 nm, were due to basal reflections indexed to a hexagonal crystal lattice with rhombohedral 3R symmetry. These values, together with other non-basal spacing at 0.152 nm (*d*_110_) and 0.149 nm (*d*_113_), were consistent with the typical XRD pattern of LDH minerals. Small broadened peaks were seen at at 2*θ* = 14.10° and 28.18°, indicating the presence of co-precipitated poorly-crystalline boehmite (CDD, PDF-2, 21-1307). The addition of AD to the AlMg−LH host caused a slight shifting of the peak (003) towards the lower angles of 2 theta. As a result, the interlayer distances of (*d*_003_) increased from 0.758 to 0.766 nm for AlMg−LH/AD 20%. The reason for this could be the strong affinity of carboxylic acid groups (−COO^−^) to magnesium ions (Mg^2+^), which were located in the inner sheets of the AlMg−LH matrix.

**Figure 5:**
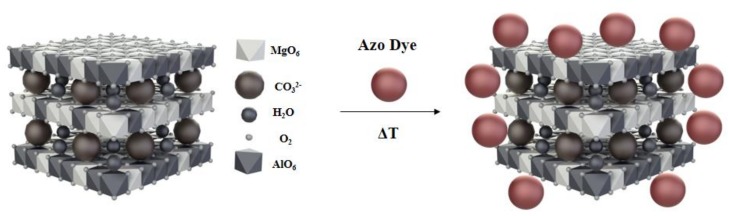
Possible structural arrangement of azo chromophore in AlMg−LH structure.

On more detailed inspection, the diffractogram of the AlMg−LH/AD 20% sample (Figure 4c) reveals a series of new peaks that did not match either pure AlMg−LH or the AD dye. At the start of this diffraction pattern, a small well-defined peak at low 2*θ* could be observed, corresponding to an expanded basal spacing of ~1.88 nm. This suggests the appearance of a new crystalline phase in the system. This phase may be related to the partial incorporation of AD into the layered structure of AlMg−LH, and to the formation of a new organic–inorganic crystalline structure. The mechanism for the formation of AlMg−LH pigments seems similar to that for lake pigments, which are usually obtained by the precipitation of dye acid structures with salts of alkaline earth [32]. The possible arrangement of the azo dye molecules in the AlMg−LH host is shown in Figure 5.

### 2.4. Morphology of Hybrid Pigment (SEM/STEM)

**Figure 6:**
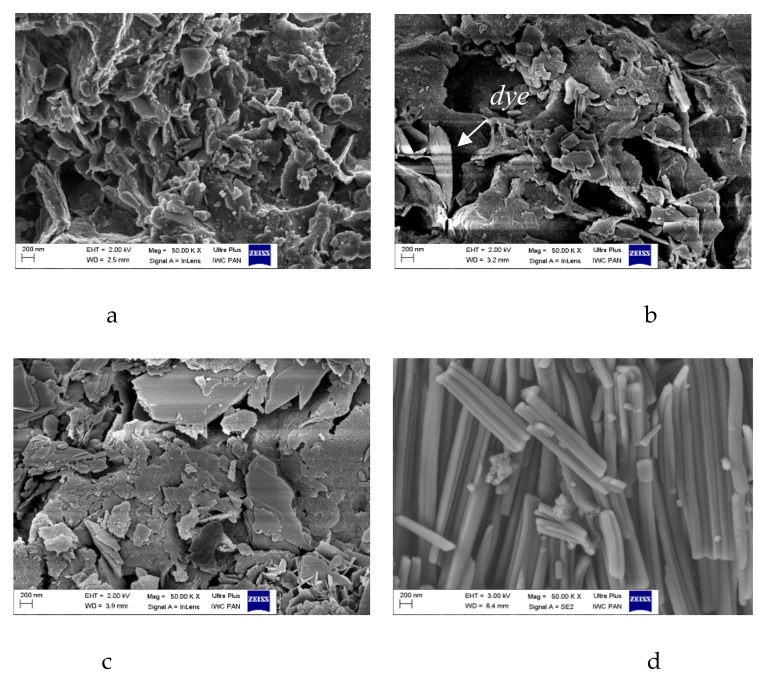
SEM micrographs of AlMg−LH (**a**), AlMg−LH/AD 10% (**b**), AlMg−LH/AD 20% (**c**), and AD (**d**).

**Figure 7:**
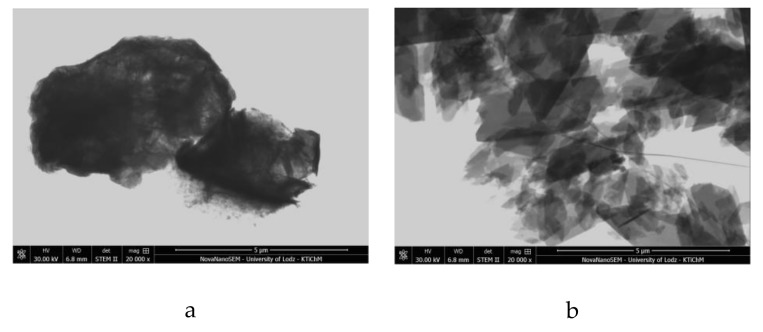

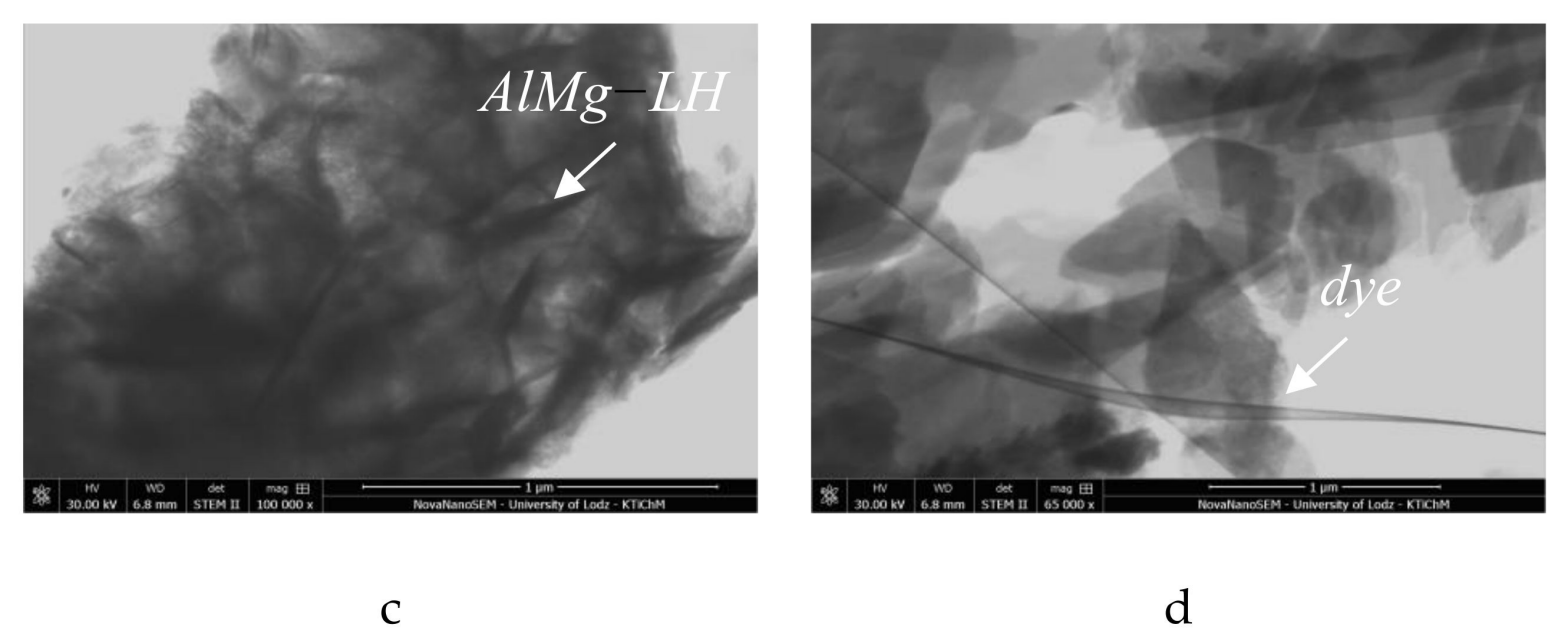
Scanning transmission electron microscope STEM micrographs of AlMg−LH (**a**,**c**) and AlMg−LH/AD 10% (**b**,**d**).

The SEM micrographs of AlMg−LH show irregular layered structures with lateral dimensions of 300–700 nm (Figure 6a–c), approximately 10–30 nm in thickness. After modification, the brick-like crystals of the azo chromophore seemed to be bound to the surface of the AlMg−LH. Based on the micrographs, the sizes of the new crystallites varied from 200 to 300 nm, and occasionally extended to 1 µm. Modification changed both the size and shape of the dye crystals (Figure 6d). This observation correlates with XRD results and can be explained by the formation of new organic–inorganic crystal structures. The STEM image in Figure 7 provides insight into the structure and agglomeration of crystals within the dye-modified AlMg−LH adsorbent. The dark lines in Figure 7a,c show the LH-layered structure (marked by an arrow). As can be seen, large numbers of dye crystals were stacked on the AlMg−LH surface after stabilization.

### 2.5. Solvent Resistance

**Figure 8:**
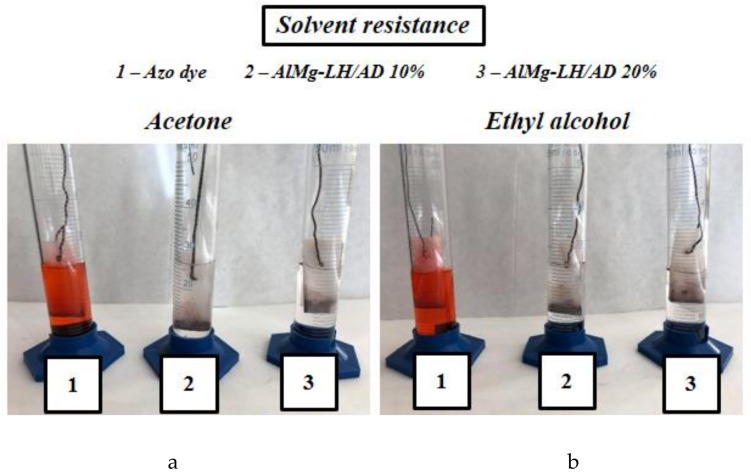
Digital images of AD dye, AlMg−LH/AD10%, and AlMg−LH/AD 20% after 24 h of immersion in acetone (**a**) and ethyl alcohol (**b**).

To evaluate their resistance to dissolution, the organic–inorganic pigments were immersed in five different solvents. The degree of discoloration was estimated after 24 h on a scale of 1–5, where 5 refers to colorless solvent (total insolubility) and 1 to intense solvent colorization (high solubility). The results in Table 1 show that the hybrid pigments exhibited excellent resistance to dissolution, while the azo dye turned the tested solvents an intense red color after immersion (Figure 8). The significantly improved solvent resistance of the azo chromophore is most likely related to fact that both carboxyl groups were involved in the formation of the organic–inorganic pigments.

### 2.6. Thermal Analysis (TGA) and Photostability of Hybrid Pigments

**Figure 9:**
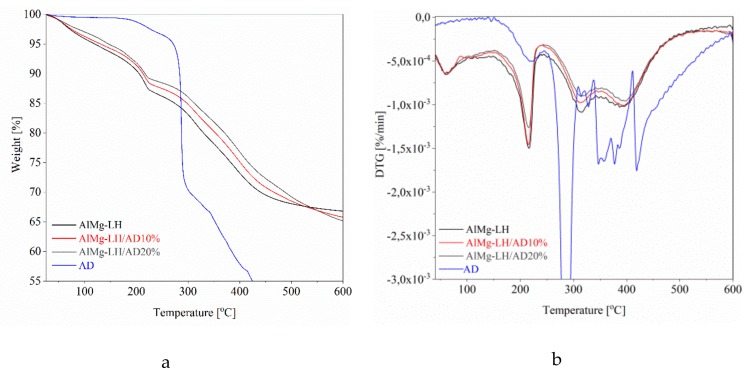
Thermogravimetric analysisTGA (**a**) and difference thermogravimetric analysis DTG(**b**) for hybrid pigments with 10% and 20% dye content.

General regions of mass loss can be observed in the TGA curves and three mass loss peaks in the DTG thermograms of the modified and unmodified samples. The first characteristic weight loss peak below 100 °C corresponded to the loss of physically absorbed water. The second mass loss region with a peak at 215 °C corresponded to the removal of water molecules from the interlayer galleries. The last weight loss stage visible at around 300–500 °C was related to the dehydration of hydroxyl groups in the brucite-like layer, as well as to the complete removal of the interlayer carbonate anions (decarbonization) [38]. The complexation of the AlMg−LH host with organic chromophore contributed to improve the thermal stability of the AlMg−LH hybrids (Figure 9). This effect was also dependent on the content of the azo dye, as can be seen by comparing the TGA curves of the different samples.

Table 2 provides a more detailed overview of the results for thermal stability. Compared to neat AlMg−LH, the *T_05%_* values of the hybrid pigments rose with increases in the quantity of AD dye. The *T_05%_* values for AlMg−LH/AD 10% and AlMg−LH/AD 20% increased from 119 °C, for pure AlMg−LH, to 131 and 145 °C, respectively. Two main weight loss peaks, at 221 and 280 °C, on the DTG curve of the AD dye were located at the same level as the dehydroxylation and carbonate decomposition peaks of the AlMg−LH matrix (Figure 9). For that reason, these peaks were absent on the TGA-DTG thermograms for the hybrid pigments (Figure 9). The weight loss temperatures of the AlMg−LH hybrids, at over 300 °C, were significantly higher than in the case of neat AlMg−LH. Weight loss temperatures in the range of 300–500 °C were related to the liberation of hydroxyl groups and carbonate ions from the layered mineral. It was assumed that carbonate ions from the weak carbonate acid were displaced from the edge of the AlMg−LH interlayer by the acidic dye. These results were consistent with TOF-SIMS measurements, in which a decrease was also noticed in the concentration of carbonate ions after modification (Figure 2). The TGA data demonstrate that incorporating the azo chromophore into the AlMg−LH structure improved the thermal stability of the organic–inorganic pigments. This was most likely due to the high thermal stability of the dye, as well as to strong dye–metal interactions between the AlMg−LH and the AD chromophore.

**Figure 10:**
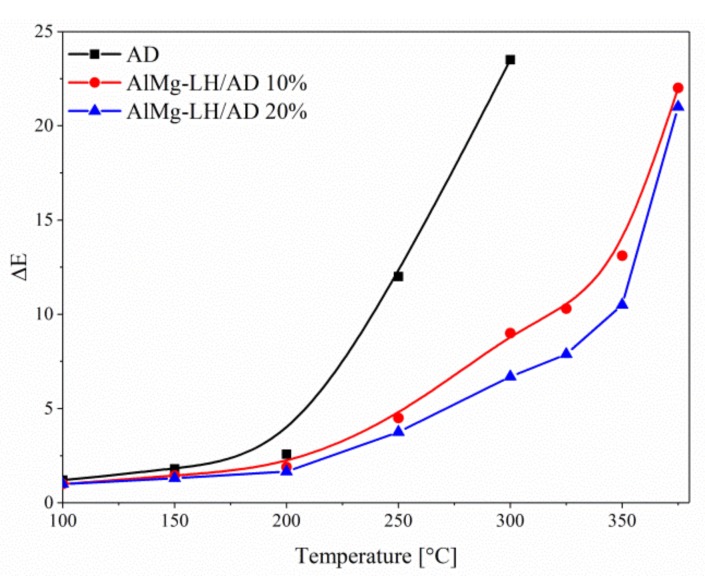
Color difference (ΔE) values for AD, AlMg−LH/AD 10%, and AlMg−LH/AD 20% powders during temperature treatment.

**Figure 11:**
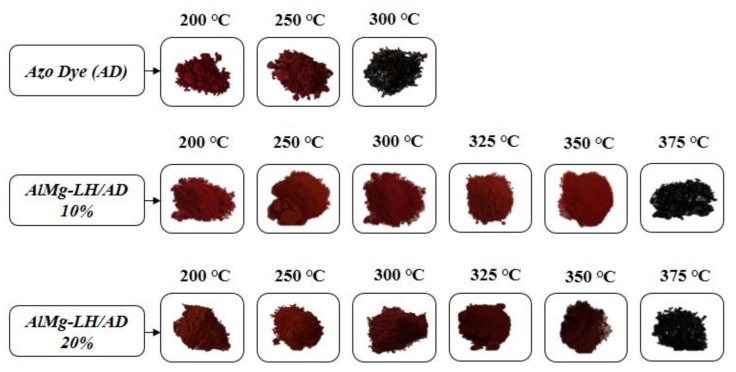
Color changes of azo dye, AlMg−LH/AD 10%, and AlMg−LH/AD 20% powders after thermal aging at different temperatures.

**Figure 12:**
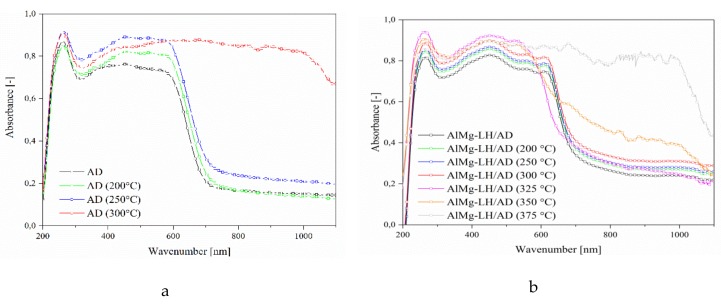
UV–VIS spectra of azo dye and hybrid pigment exposed to different temperatures: (**a**) AD; and (**b**) AlMg−LH/AD 20%.

Samples of the AD dye, AlMg−LH/AD 10% and AlMg−LH/AD 20% were heated in an oven at 100, 150, 200, 250, 300, 325, 350, and 375 °C for 30 min. The treated pigments were measured using the CIE 1976 L*a*b* method. The results were expressed in terms of the total color difference ∆E (Figure 10) and differences in the color parameters L*, a*, b* (Table S1, Supplementary data). The ΔE value of the pure dye rose from 5 (200 °C) to 20 after treatment at 250 °C, indicating that the non-modified dye underwent thermal decomposition at this temperature. The color variation of the AlMg−LH/AD 20% pigment was significantly lower than that of the pure dye after temperature treatment for the same length of time (Figure 11). This observation was confirmed by the diffuse reflectance UV–VIS spectra of the resulting pigments (Figure 12). The spectra of the dye underwent marked variation after heating at 300 °C, especially in the range of 400–700 nm. However, in the case of AlMg−LH/AD 20% there were no significant changes in the spectra after heating at any of the considered temperatures. When the samples were heated at 300 °C, the azo dye became carbonized and turned black, as shown in Figure 11. In contrast, the AlMg−LH/AD 10% and AlMg−LH/AD 20% pigments showed a much more limited color change, indicating better thermostability. Decomposition was observed at 375 °C. The ΔE parameter was slightly higher for AlMg−LH/AD 10% pigment (Figure 10) and more pronounced changes were observed in the absorbance spectra (Figure S1), most likely due to a lower concentration of azo dye being incorporated into the inorganic host. This demonstrates, conclusively, that the enhanced thermostability of the dye in the layered mineral was due mainly to strong host–guest interactions (AlMg−LH/AD).

**Figure 13:**
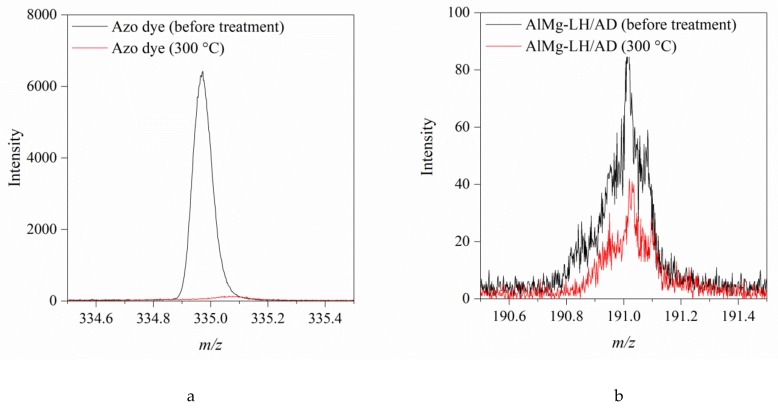
TOF-SIMS spectra of C_18_H_11_O_5_N_2_^−^ (**a**) and C_18_H_10_O_5_N_2_Mg_2_^2+^ ions (**b**) from the AlMg−LH/AD 20% sample before and after heating at 300 °C.

The TOF-SIMS technique was also employed to monitor the thermal resistance of the azo dye and its complexes in the AlMg−LH/AD 10% and AlMg−LH/AD 20% samples. Figure 13a shows the complete disappearance of the peak characteristic for AD after annealing of the sample at 300 °C. The characteristic peak of the C_18_H_10_O_5_N_2_Mg_2_^2+^ ion in the TOF-SIMS spectrum of the AlMg−LH/AD 20% sample annealed at 300 °C indicates that interaction between the AD and the AlMg−LH host resulted in a hybrid with improved color stability (Figure 13b). Hybrid pigment modified with 10% dye showed a less intense characteristic peak for the C_18_H_10_O_5_N_2_Mg_2_^2+^ ion after annealing at 300 °C. However, the decomposition trend was similar to that of the AlMg−LH/AD 20% sample (Figure S2). These results are in line with spectrophotometric studies (CIEL*a*b* and UV–VIS), demonstrating that TOF-SIMS can be a complementary method for monitoring the stability of such complexes.

**Figure 14:**
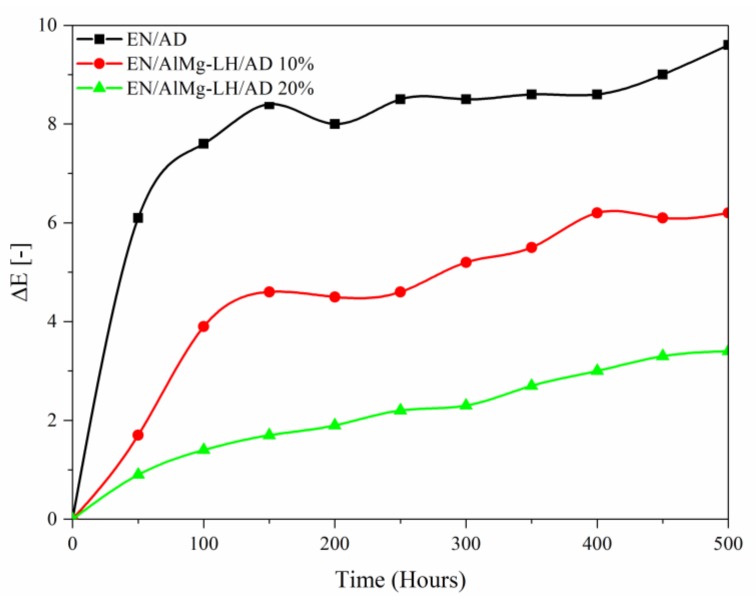
Color difference (ΔE) values for ethylene–norbornene (EN) composites filled with AD and AlMg−LH/AD pigments during UV aging.

The AlMg−LH/pigments (10% and 20% AD) and azo dye were used as colorants for the ethylene–norbornene copolymer and exposed to UV light irradiation (Figure 14 and Figure S3) The color difference (ΔE) values of the irradiated samples were measured every 50 h, up to a total exposure time of 500 h. It was noted that composites containing organic–inorganic pigments exhibited higher photostability to UV light than the neat chromophore. The ΔE value of the dye increased up to 8 when the aging time reached 200 h, indicating that the non-modified dye had undergone photooxidation. The color changes of the AlMg−LH/AD 20% pigment were significantly weaker than those of the pure dye after irradiation for the same length of time. For the AlMg−LH/AD 10% pigment, the total value for color difference was half that of AD after irradiation for 300 h. These results indicate that, as well as increasing the heat resistance of the metal–dye hybrid, modification with AD also contributed to improve the light resistance of the studied chromophore.

## 3. Experimental

### 3.1. Materials

Aluminum–magnesium hydroxycarbonate (AlMg−LH, Mg:Al weight ratio 30:70) with the empirical formula Mg_0.33_Al_0.67_(OH)_2_(CO_3_)_0.33_ nH_2_O was supplied by Sasol (Germany). Anthranilic acid (98%), 3-hydroxy-2-naphthoic acid, hydrochloric acid (37%), acetic acid (99.8%), ethyl alcohol (95%), acetone (99.9%), n-butyl acetate (99.9%), and toluene (99.9%) were purchased from Sigma-Aldrich (Germany). Ethylene–norbornene random copolymer (EN) was employed as the polymer matrix, supplied by TOPAS Advanced Polymers (Germany). The synthesis of carboxylic azo dye (4-[2-(2-Carboxyphenyl)diazenyl]-3-hydroxy-2-naphthalenecarboxylic acid) was carried out according to the procedure described in the next section.

#### 3.1.1. Azo Dye Synthesis

**Figure 15:**
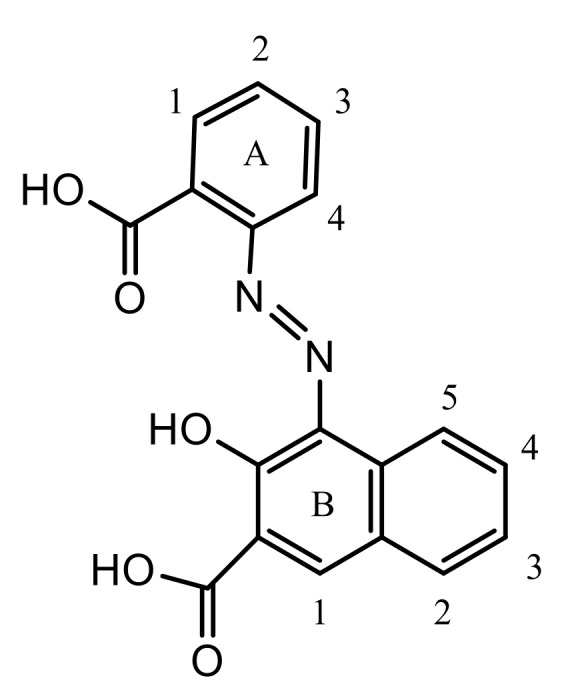
Structural formula of studied azo dye (AD).

Dicarboxylic azo dye was synthesized following the typical diazotization procedure with some minor changes (Figure 15) [39]. First, 14 g of anthranilic acid (0.1 mol) was dissolved in 100 ml of deionized H_2_O and 10 cm^3^ of 30% NaOH solution. Next, 40 cm^3^ of 30% hydrochloric acid was added and the mixture was cooled to below 10 °C. Finally, ice cold aqueous NaNO_2_ solution (25 cm^3^) was used for diazotization. The reaction mixture was stirred for 1 h at 0–5 °C. Completion of diazotization was verified using starch-iodide paper.

In the coupling step, a solution of 3-hydroxy-2-naphthoic acid (0.1 mol, 18.8 grams) was prepared in 150 cm^3^ of deionized water and 30 cm^3^ of 30% NaOH. The reaction mass was cooled to 0–5 °C, and a previously-prepared diazonium salt solution was added dropwise with continuous stirring and the temperature maintained at 0–5 °C. A color reaction with H-acid was used to confirm the reaction endpoint. The product of the coupling reaction was acidified with 20 cm^3^ of 30% hydrochloric acid. Finally, the separated dye was filtered, washed several times with water, and dried at 60–65 °C. The crude product was recrystallized from acetic acid (yield: 68%). ^1^H-NMR, FT-IR, and UV–VIS spectra of the prepared azo dye are presented in Figures S4 and S5.

^1^H-NMR (DMSO-d_6_, 250 MHz) δ_H_: 8.37 (d, 1H, (A) H-1), 7.75 (m, 2H, (A) H-2), 7.68 (m, 2H, (A) H-3), 8.45 (d, 1H, (A) H-4), 8.62 (s, 1H, (B) H-1), 7.92 (d, 1H, (B) H-2), 7.36 (t, 2H, (B) H-3), 7.51 (m, 2H, (B) H-4), 8.04 (d, 1H, (B) H-5), 13.53 (COOH), 3.29 (OH). TOF-SIMS Calcd for C_18_H_11_O_5_N_2_^−^ [M-H]^−^, *m/z* = 335.075; Found, *m/z* = 335.074.

#### 3.1.2. Hybrid Pigment Preparation

Modification was carried out for samples with 10% and 20% AD content. In this section, the AlMg−LH/AD 10% sample is taken as an example. First, 1 g (for AlMg−LH/AD 20% sample 2 g) of synthetic azo dye was dissolved in an aqueous solution (200 cm^3^ of deionized water) with the addition of 10 cm^3^ of ethanol. The sample was subjected to ultrasonication for 30 min. The solution was then heated and 9 g (for AlMg−LH/AD 20% sample 8 g) of AlMg−LH was added. The reaction mixture was kept at 80 °C for 3 h with stirring, after which the precipitated pigment slurry was filtered under a vacuum and washed repeatedly with deionized water until a colorless solution was observed. Finally, the hybrid pigment powder was dried in an oven at 75 °C for 24 h.

The amount of the organic chromophore stabilized by the AlMg−LH matrix was measured using N elemental analysis (Table S2, Supplementary Material). The largest increase in N concentration was observed for the sample modified with 10% azo chromophore, where the found (0.84) and calculated (0.84) nitrogen were at the same level. The modification with 20% of dye resulted in a lower N concentration of approximately 1.58, while expected concentration was around 1.67.

#### 3.1.3. Preparation of Ethylene-Norbornene (EN) Composites

The ethylene–norbornene composites were prepared using a Brabender measuring mixer N50 (Duisburg, Germany) at 120–130 °C. Homogenization of the EN/hybrid pigments was continued for 10 min. After the mixing process, the compounds were sheeted between two steel plates at 120 °C for 10 min. Composites were prepared using the following formulation (in parts per hundred parts of rubber—phr): ethylene–norbornene copolymer (100 phr) and hybrid pigment (3 phr).

### 3.2. Characterization

Nuclear magnetic resonance (NMR) was performed to confirm the structures of the synthesized carboxylic dyes. The ^1^H-NMR spectra were recorded on a Bruker Avance DPX (Rheinstetten, Germany) 250 MHz instrument, using DMSO-d6 as solvent.

Solid state Nuclear Magnetic Resonance (MAS NMR) measurements were performed in a Bruker Avance III 400 WB (Rheinstetten, Germany) spectrometer operating at a resonance frequency of 104.26 MHz. ^27^Al chemical shifts were referenced using AlCl_3_·6H_2_O in 1M solution as an external reference (0 ppm).

Secondary ion mass spectra were recorded using a TOF-SIMS IV mass spectrometer (ION-TOF GmbH, Muenster, Germany). This instrument is equipped with a Bi liquid metal ion gun as well as a high mass resolution time of flight mass analyzer. The measuring area covered 100 × 100 μm of the sample surface and the analysis time was 30 s. During analysis, the area of the tested sample was irradiated with pulses of 25 keV Bi_3_^+^ ions at a 10 kHz repetition rate and with an average ion current of 0.4 pA.

X-ray powder diffraction patterns were collected using a PANalytical X’Pert Pro MPD diffractometer in the Bragg–Brentano reflection geometry with (CuK_α_) radiation from a sealed tube (Malvern Panalytical Ltd., Royston, UK). The apparatus operates in the range of 2*θ* = 2–70° with a step size of 0.0167°.

UV–Visible absorption measurement were obtained using an Evolution 201/220 UV–Visible Spectrophotometer (Thermo Scientific, Waltham, MA, USA), with a spectral window from 1100 to 200 nm. The measurement specimens were in the form of solid-state powders. They were placed in a special powder cell holder. Before the measurements, the baseline was corrected using a special calibration adapter. The accuracy of the apparatus was ± 0.8 nm and the repeatability was ≤ 0.05 nm.

The UV–Visible absorption of the pure dye was also studied in chloroform (dye concentration 1 × 10^−4^ M) using an Evolution 201/220 UV–Visible Spectrophotometer (Thermo Scientific, Waltham, MA, USA) and standard quartz cuvettes. The absorption spectra were recorded across a wavelength range of 1100–200 nm.

Thermal stability (annealing) of hybrid pigments and AD dye was performed in an oven under a static air atmosphere (Binder, Tuttlingen, Germany). The samples were heated in an oven at 100, 150, 200, 250, 300, 325, 350, and 375 °C for 30 min.

The FT-IR spectra were recorded using a Thermo Scientific Nicolet 6700 FT-IR spectrometer (Waltham, MA, USA) with a resolution of 4 cm^−1^. The measurements were performed at room temperature across a wave number range 600–4000 cm^−1^ (64 scans).

Elemental analyses of carbon, hydrogen, and nitrogen in the hybrid pigment powders were carried out using a Vario EL III analyzer equipped with special adsorption columns and a thermal conductivity detector (TCD).

Thermogravimetry and differential thermal analysis (TGA-DTG) were performed with a TA Instruments Q500 Thermogravimetric Analyzer (TA Instruments, Greifensee, Switzerand). Measurements were conducted in the presence of argon, in a temperature range of 25–600 °C, with a heating rate of 10 °C/min.

The resistance of the hybrid pigments to organic solvents was determined based on the PN-C-04406/1998 standard. The powders (about 0.05 g) were immersed in various solvents (water, toluene, acetone, n-butyl acetate, and ethyl alcohol) for 24 h at room temperature. After this period, their degree of decolorization was assessed.

Accelerated weathering of the pigments was performed using a UV2000 Atlas solar simulation chamber (Atlas, Linsengericht, Germany). The measuring setup included day conditions (radiation intensity 0.7 W/m^2^, temperature 60 °C, duration 8 h) and night conditions (no UV radiation, temperature 50 °C, duration 4 h). In total, the simulation lasted 500 h. The samples were inspected every 50 h.

The color change (ΔE) in the materials aged under UV light was determined in terms of the CIE L* a* b* color space system using a CM-3600d spectrophotometer (Konica Minolata Sensing Inc., Osaka, Japan) with a spectral range of 360–740 nm. The color measurements were carried out on a white calibration plate. The total color difference was calculated using the equation:

(1) $$\Delta E=\sqrt{{\left(\Delta L\right)}^{2}+{\left(\Delta a\right)}^{2}+{\left(\Delta b\right)}^{2}}$$

where ∆L—level of lightness or darkness, ∆a—relationship between redness and greenness, and ∆b—relationship between blueness and yellowness.

Scanning electron microscopy (SEM) analysis was performed on a Leo 1530 Gemini scanning electron microscope (Zeiss/Leo, Oberkochen, Germany). The pigment powders were coated with a carbon target using a Cressington 208 h system. The structures of the pigments were also analyzed using a high-resolution scanning transmission electron microscope (STEM) (FEI, NovaNanoSEM 450; accelerating voltage 30 kV). Samples for STEM measurements were prepared by depositing the colloids onto carbon-coated copper grids.

## 4. Conclusions

In this study, a new type of hybrid pigment was created by the complexation of dicarboxylic azo dye with an aluminum–magnesium hydroxycarbonate inorganic host. The presence of C_18_H_10_O_5_N_2_Mg_2_^2+^ ions in TOF-SIMS spectra showed that the dye can be stabilized by two Mg ions due to the presence of two COO^−^ groups in dye molecule. Moreover, the ^27^Al MAS spectra of the hybrid pigments revealed possible interactions of the studied dye with Al ions. Formation of the new organic–inorganic structure was confirmed by XRD analysis, as the diffraction patterns contained a set of reflections which did not belong to either AlMg−LH or the AD chromophore. The new organic–inorganic pigment showed excellent resistance to dissolution in the solvents: acetone, ethyl alcohol, and toluene. A further advantage is their considerably improved thermal and photostability, which are suitable for a wider range of polymer applications. Secondary ion mass spectrometry was found to be a valuable technique for investigating the interactions between the organic chromophore and particular components in the inorganic matrix, including Mg^2+^ ions. Such observations are not possible using more commonly-used methods, such as NMR spectroscopy. The results of TOF-SIMS studies proved that this technique can also be considered a useful tool for analyzing the thermal stability of hybrid colorants.

## Figures and Tables

**Table 1:** Solvent resistance of hybrid pigments.

Hybrid Pigment	Solvent Resistance				
	Water	Acetone	Toluene	Ethanol	Butyl Acetate
AlMg−LH/AD 10%	5	5	5	5	5
AlMg−LH/AD 20%	5	5	5	5	5

**Table 2:** Thermogravimetric analysis of AlMg−LH/AD pigments.

Sample	*T_05_^1^* (°C)	*T_10_^1^* (°C)	*T_20_^1^* (°C)	*T_30_^1^* (°C)
AlMg−LH	119	205	330	441
AlMg−LH/AD 10%	131	214	353	466
AlMg−LH/AD 20%	145	217	370	488

^1^ T_05_, T_10_, T_20_, T_30_—temperature of 5%, 10%, 20%, and 30% weight loss.

## Supplementary Materials

The following are available online. Figure S1. UV–VIS spectra of hybrid pigment (AlMg−LH/AD 10%) exposed to different temperatures. Figure S2. TOF-SIMS spectra of the C18H10O5N2Mg22+ ion from the AlMg−LH/AD 10% sample before and after heating at 300 °C. Figure S3. Digital photographs of ethylene–norbornene (E–N) composites: E–N, E–N/AlMg−LH/AD 10% and AlMg−LH/AD 20% before and after 500 h of UV aging. Figure S4. 1H-NMR spectrum of AD dye. Figure S5. Absorption spectra of AD dye in chloroform (c = 1 × 10−4 M) (a), and FT-IR spectra of AD dye (b). Table S1. Color parameters of AD dye and hybrid pigment AlMg−LH/AD 20% during temperature treatment. Table S2. Elemental composition of the hybrid pigments.

## References

[1] Tang P., Feng Y., Li D. (2012). Improved thermal and photostability of an anthraquinone dye by intercalation in a zinc-aluminum layered double hydroxides host. Dyes Pigm..

[2] Liu P., Liu P., Zhao K., Li L. (2015). Photostability enhancement of azoic dyes adsorber and intercalated into Mg-Al-layered double hydroxide. Opt. Laser. Technol..

[3] Pardo R., Zayat M., Levy D. (2011). Photochromic organic–inorganic hybrid materials. Chem. Soc. Rev..

[4] Tang P., Feng Y., Li D. (2014). Facile synthesis of multicolor organic–inorganic hybrid pigments based on layered double hydroxides. Dyes Pigm..

[5] Shi W., He S., Wei M., Evans D., Duan X. (2010). Optical pH sensor with rapid response based on a fluorescein-intercalated layered double hydroxide. Adv. Funct. Mater..

[6] Latterini L., Nocchetti M., Aloisi G.G., Costantino U., Elisei F. (2007). Organized chromophores in layered inorganic matrices. Inorg. Chim. Acta..

[7] Martinez-Zapata O., Mendez-Vivar J., Bosch P., Lara VH. (2011). Synthesis and characterization of amorphous aluminosilicates prepared by sol-gel to encapsulate organic dyes. J. Non-Cryst Solids.

[8] Perez E., Lima E., Guzman A. (2015). Natural betalains supported on γ-alumina: A wide family of stable pigments. Dyes Pigm..

[9] Jesionowski T. (2005). Characterisation of pigments obtained by adsorption of CI Basic Blue 9 and CI Acid Orange 52 dyes onto silica particles precipitated via the emulsion route. Dyes Pigm..

[10] Loera S., Ibarra I.A., Laguna H., Lima E., Bosch P., Lara V., Haro-Poniatowski E. (2006). Colored sodalite and A zeolites. Ind. Eng. Chem. Res..

[11] Maas H., Khatyr A., Calzaferri G. (2003). Phenoxazine dyes in zeolite L, synthesis and properties. Microporous Mesoporous Mater..

[12] Hwang S.H., Jung S.C., Yoon S.M., Kim D.K. (2008). Preparation and characterization of dye-intercalated ZnAl-layered double hydroxide and its surface modification by silica coating. J. Solid State Chem..

[13] Kohno Y., Totsuka K., Ikoma S., Yoda K., Shibata M., Matsushima R., Tomita Y., Maeda Y., Kobayashi K. (2009). Photostability enhancement of anionic natural dye by intercalation into hydrotalcite. J. Colloid Interface Sci..

[14] Maruyama S.A., Tavares S.R., Leitao A.A., Wypych F. (2016). Intercalation of indigo carmine anions into zinc hydroxide salt: a novel alternative blue pigment. Dyes Pigm..

[15] Bourhis K., Blanc S., Mathe C., Dupin JC., Vieillescazes C. (2011). Spectroscopic and chromatographic analysis of yellow flavonoidic lakes: quercitin chromophore. Appl. Clay. Sci..

[16] Doskocz M., Kubas K., Frackowiak A., Gancarz R. (2009). NMR and ab initio studies of Mg^2+^, Ca^2+^, Zn^2+^, Cu^2+^ alizarin complexes. Polyhedron.

[17] Deveoglu O., Cakmakc E., Taskopru T., Torgan E., Karadag R. (2012). Identification by RP-HPLC-DAD, FTIR, TGA and FESEM-EDAX of natural pigments prepared from *Datisca cannabina*L.. Dyes Pigm..

[18] Hussein M.Z., Yahaya A.H., Ping L.M. (2004). Dye-interleaved nanocomposite: Evan’s Blue in the lamella of Mg-Al-layered double hydroxide. Dyes Pigm..

[19] Guillermin D., Debroise T., Trigueiro P., de Viguerie L., Rigaud B., Morlet-Savary F., Balme S., Janot J.M., Tielens F., Michot L. (2019). New pigments based on carminic acid and smectites: A molecular investigation. Dyes Pigm..

[20] Perez E., Ibarra I.A., Guzman A., Lima E. (2017). Hybrid pigments resulting from several guest dyes onto γ-alumina host: A spectroscopic analysis. Spectrochim. Acta A..

[21] Girdthep S., Sirirak J., Daranarong D., Daengngern R., Chayabutra S. (2018). Physico-chemical characterization of natural lake pigments obtained from Caesalpinia Sappan Linn. and their composite films for poly(lactic acid)-based packaging material. Dyes Pigm..

[22] Marangoni R., Ramos L.P., Wypych F. (2009). New multifunctional materials obtained by the intercalation of anionic dyes into layered zinc hydroxide nitrate followed by dispersion into poly(vinyl alcohol)(PVA). J. Colloid. Interface Sci..

[23] Tang P., Xu X., Lin Y., Li D. (2008). Enhancement of the thermo- and photo-stability of an anionic dye by intercalation in a zinc-aluminum layered double hydroxide host. End. Eng. Chem. Res..

[24] Chakraborty C., Dana K., Malik S. (2010). Intercalation of perylenediimde dye into LDH clays: enhancement of photostability. J. Phys. Chem. C..

[25] Cavani F., Trifirb F., Vaccari A. (1991). Hydrotalcite-type anionic clays: Preparation, properties and applications. Catal. Today.

[26] Evans D.G., Duan X. (2006). Preparation of layered double hydroxides and their applications as additives in polymers, as precursors to magnetic materials and in biology and medicine. Chem. Commun..

[27] Forano C., Costantino U., Prevot V., Taviot-Gueho C. (2006). Handbook of Clay Science.

[28] Sun Z., Jin L., Shi W., Wei M., Duan X. (2010). Preparation of an anion dye intercalated into layered double hydroxides and its controllable luminescence properties. Chem. Eng. J..

[29] Bauer J., Behrens P., Speckbacher M., Langhals H. (2003). Composites of perylene chromophores and layered double hydroxides: direct synthesis, characterization, and photo- and chemical stability. Adv. Funct. Mater..

[30] Kutlu B., Leuteritz A., Häußler L., Oertel U., Heinrich G. (2014). Stabilization of polypropylene using dye modified layered double hydroxides. Polym. Degrad. Stab..

[31] Kennedy A.R., Stewart H., Eremin K., Stenger J. (2012). Lithol red: A systematic structural study on salts of a sulfonated azo pigment. Chem. Eur. J..

[32] Christie R.M., Mackay J.L. (2008). Metal salt azo pigments. Color. Technol..

[33] Marzec A., Szadkowski B., Rogowski J., Maniukiewicz W., Moszyński M., Kozanecki M., Zaborski M. (2018). Characterization and properties of new color-tunable hybrid pigments based on layered double hydroxides (LDH) and 1,2-dihydroxyanthraquinone dye. J. Ind. Eng. Chem..

[34] Fournier F., Viguerie L., Balme S., Janot J.M., Walter P., Jaber M. (2016). Physico-chemical characterization of lake pigments based on montmorillonite and carminic acid. Appl. Clay. Sci..

[35] Park T.J., Choi S.S., Kim Y. (2009). ^27^Al solid-state NMR structural studies of hydrotalcite compounds calcined at at different temperatures. Bull. Korean Chem. Soc..

[36] Trigueiro P., Pereira F.A.R., Guillermin D., Rigaud B., Balme S., Janot J.M., Santos I.M.G., Fonseca M.G., Walter P., Jaber M. (2018). When anthraquinone dyes meet pillared montmorillonite: Stability or fading upon exposure to light?. Dyes Pigm..

[37] Sharma S.K., Kushwaha P.K., Srivastava V.K., Bhatt S.D., Jasra R.V. (2007). Effect of hydrothermal conditions on structural and textural properties of synthetic hydrotalcites of varying Mg/Al ratio. Ind. Eng. Chem. Res..

[38] Costa F.R., Leuteritz A., Wagenknecht U., Jehnichen D., Häußler L., Heinrich G. (2008). Intercalation of Mg-Al layered double hydroxide by anionic surfactants: preparation and characterization. Appl. Clay Sci..

[39] Tewari A.K., Mishra A. (2006). Synthesis and antiviral activities of N-substituted-2-substituted benzimidazole derivatives. Indian J. Chem. Sec. B..

