# Supplementary material for: Characterization and Structure–Property Relationships of Organic–Inorganic Hybrid Composites Based on Aluminum–Magnesium Hydroxycarbonate and Azo Chromophore
Source: Molecules. 2019 Mar 1;24(5):880. doi: 10.3390/molecules24050880 (PMC6429098; doi:10.3390/molecules24050880)
Supplement: Supplementary file 1 [file molecules-24-00880-s001.pdf]

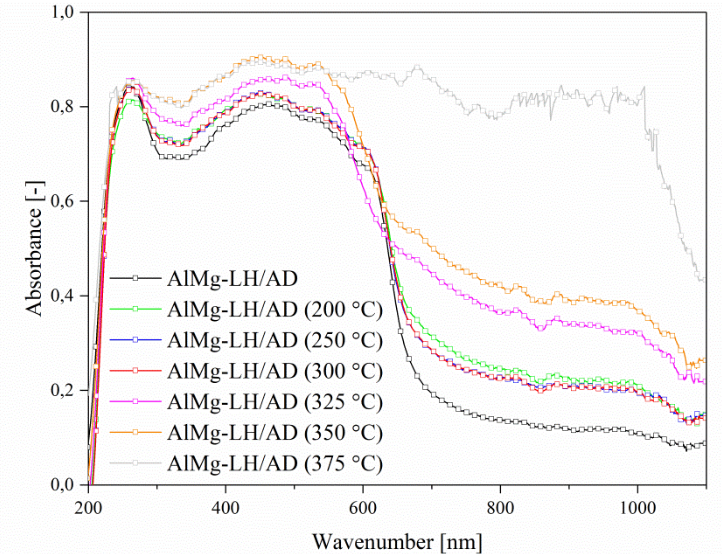

Figure S1. UV–VIS spectra of hybrid pigment (AlMg–LH/AD 10%) exposed to different temperatures.

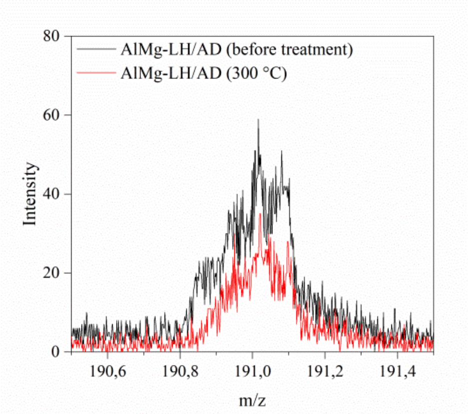

Figure S2. TOF-SIMS spectra of the $C_{18}H_{10}O_5N_2Mg_2^{2+}$ ion from the AlMg–LH/AD 10% sample before and after heating at 300 °C.

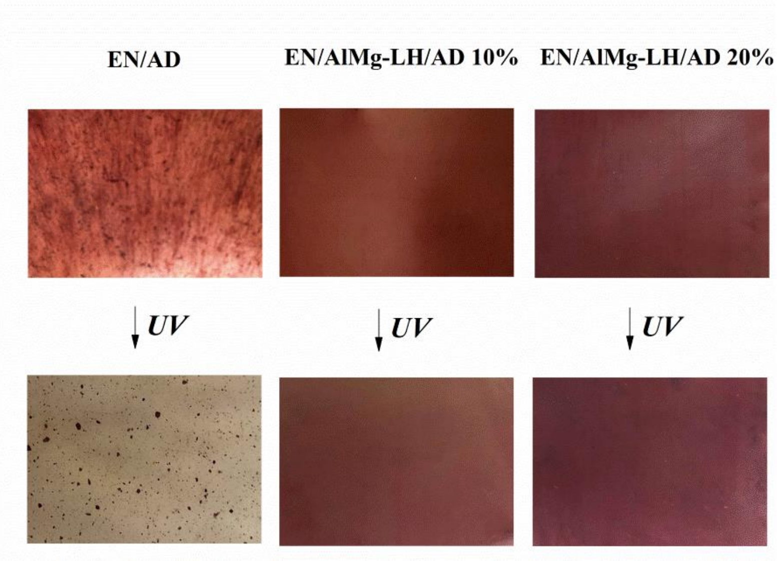

Figure S3. Digital photographs of ethylene–norbornene (E–N) composites: E–N, E–N/AlMg–LH/AD 10% and AlMg–LH/AD 20% before and after 500 h of UV aging.

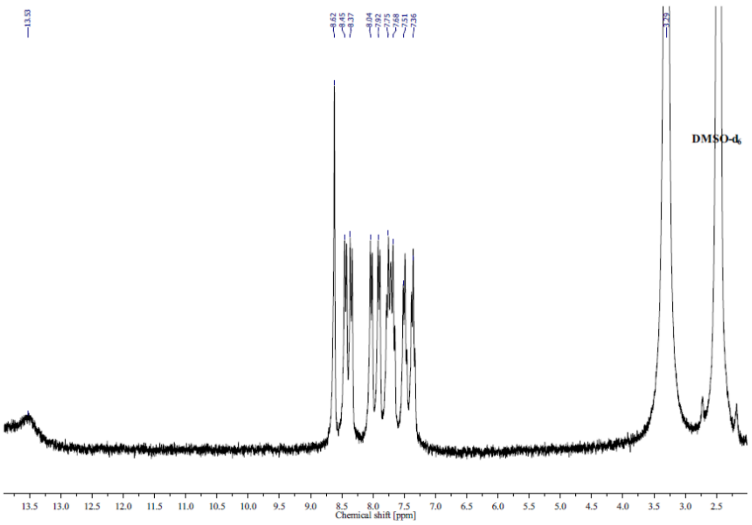

Figure S4. ^{1}H NMR spectrum of AD dye.

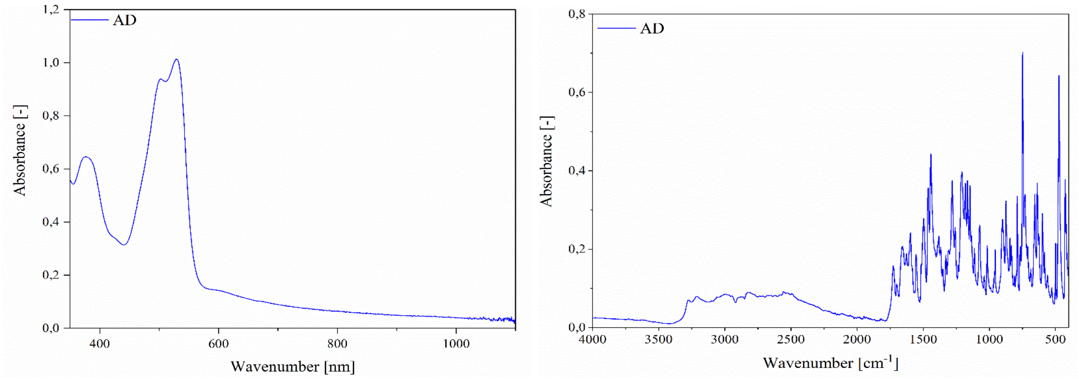

a b

Figure S5. Absorption spectra of AD dye in chloroform (c = 1×10^{-4} M) (**a**), and FT-IR spectra of AD dye (**b**).

Table S1. Color parameters of AD dye and hybrid pigment AlMg–LH/AD 20% during temperature treatment.

Sample	L^{*1}	a^{*2}	b^{*3}
AD	38.18	18.92	14.75
AD (150 ℃)	38.02	21.06	15.12
AD (200 ℃)	33.04	2.70	0.11
AD (250 ℃)	33.25	1.48	−0.11
AlMg–LH	43.66	7.18	30.59
AlMg–LH/AD (150 ℃)	42.31	8.02	30.14
AlMg–LH/AD (200 ℃)	41.02	8.41	28.23
AlMg–LH/AD (250 ℃)	40.30	11.47	26.70

[1] L^*—lightness, [2]a^*—negative values for green and positive values for red, [3]b^*—negative values for blue and positive values for yellow.

Table S2. Elemental composition of the hybrid pigments.

**Hybrid Pigment**	*%C**	*%H**	*%N**	*%N***
AlMg–LH	1.64; 1.54	3.19; 3.20	-	
AlMg–LH/AD 10%	7.18; 7.32	3.17; 3.20	0.84; 0.79	0.84
AlMg–LH/AD 20%	12.39; 12.23	3.29; 3.32	1.58; 1.59	1.67

*received, **expected.
